# Non-surgical treatment of tetanus infection associated with breast cancer skin ulcer: a case report and literature review

**DOI:** 10.1186/s12879-020-05739-4

**Published:** 2021-01-07

**Authors:** Kazuhito Nomura, Eiji Sakawaki, Sonoko Sakawaki, Ayumu Yamaoka, Wakiko Aisaka, Hiroyuki Okamoto, Yoshihiro Takeyama, Shuji Uemura, Eichi Narimatsu

**Affiliations:** 1 Department of Emergency Medicine, Sapporo Medical University, Minami 1-jo, Nishi 16-chome, 291, Chuo-ku, Sapporo-shi, Hokkaido 060-8543 Japan; 2 Department of Emergency Medicine, Hakodate Municipal Hospital, 1-chome 10-1, Minato-cho, Hakodate-shi, Hokkaido 041-8680 Japan; 3 Department of Neurosurgery, Sunagawa City Medical Center, 3-chome1-1 Nishi 4-jo Kita, Sunagawa-shi, Hokkaido 073-0196 Japan; 4 Department of Emergency Medicine, Teine Keijinkai Hospital, 12-chome 1-40, Maeda 1-jo, Teine-ku, Sapporo-shi, Hokkaido 006-0811 Japan

**Keywords:** Tetanus, chronic wound, breast cancer, skin ulcer, non-operative strategy

## Abstract

**Background:**

Previous studies have reported poor prognosis in cases of tetanus that develops after bacteria enters via breast cancer-related skin ulcers that are not treated with surgical debridement. Herein, we review the literature concerning this presentation and report the first case of complete remission from tetanus without surgical debridement of the skin ulcer.

**Case presentation:**

An Asian woman aged over 60 years had a history of skin ulcer caused by breast cancer. She was diagnosed with tetanus due to trismus and opisthotonus. Based on the suspicion that the skin ulcer was the portal of entry for tetanus bacteria, we considered several debridement and thoracic surgical options for tetanus treatment. However, debridement was not performed as the surgery was considered high risk and the patient did not consent to it. The patient received treatment with anti-tetanus globulin and metronidazole; sound insulation and shielding were also performed in a dark room. Subsequently, the patient’s symptoms improved, and sound insulation and deep sedation management were completed on 19th day of hospitalization. With no symptom recurrence, the patient was discharged on Day 54. To date, over 3 years after treatment, no evidence of tetanus recurrence has been observed. The case was characterized by a lack of autonomic hyperactivity. The tetanus severity was likely representative of the low amount of toxin that the patient was exposed to.

**Conclusion:**

This case involved moderate severity tetanus originating from a chronic skin ulcer related to breast cancer. The patient survived without undergoing extensive debridement. No evidence of tetanus relapse was observed during the follow-up period, likely due to vaccination that might have restored the patient’s active immunity. Debridement is not always necessary for tetanus complicated by breast cancer skin ulcers. Furthermore, appropriate toxoid vaccination is critical for preventing the onset and recurrence of tetanus in these patients.

## Background

Tetanus is caused by *Clostridium tetani*, an obligate anaerobic bacterium, that enters the body and releases toxins. The tetanus toxin causes severe spastic paralysis and autonomic hyperactivity, which may lead to death [1, 2]. Patients with chronic wounds such as skin ulcer are at a high risk of tetanus as these wounds provide an entry route for tetanus bacteria [3]. In fact, previous studies have shown that patients with skin ulcer due to advanced breast cancer are at a particularly high risk of developing tetanus and that removing the ulcer is the only effective treatment in such cases [4–17]. A cancerous skin ulcer contains necrotic tissue, which constitutes anaerobic environment that is conducive to the growth of tetanus bacteria and the production of toxins [3, 18, 19]. As blood flow to necrotic tissues is restricted, treatment with antibiotics tends to be ineffective in such cases due to the body’s inability to deliver the treatment to the site of infection. In addition, tetanus bacteria can form spores, which may reduce the effectiveness of antibacterial drugs. As a result, in cases of tetanus associated with chronic wounds, debridement of the wound has been considered an essential element of treatment [1, 3, 19–21]. Herein, we report about a patient with tetanus due to skin ulcer associated with breast cancer who was treated without debridement and who presented with no sign of recurrence 3 years after treatment completion.

## Case presentation

**Fig. 1:**
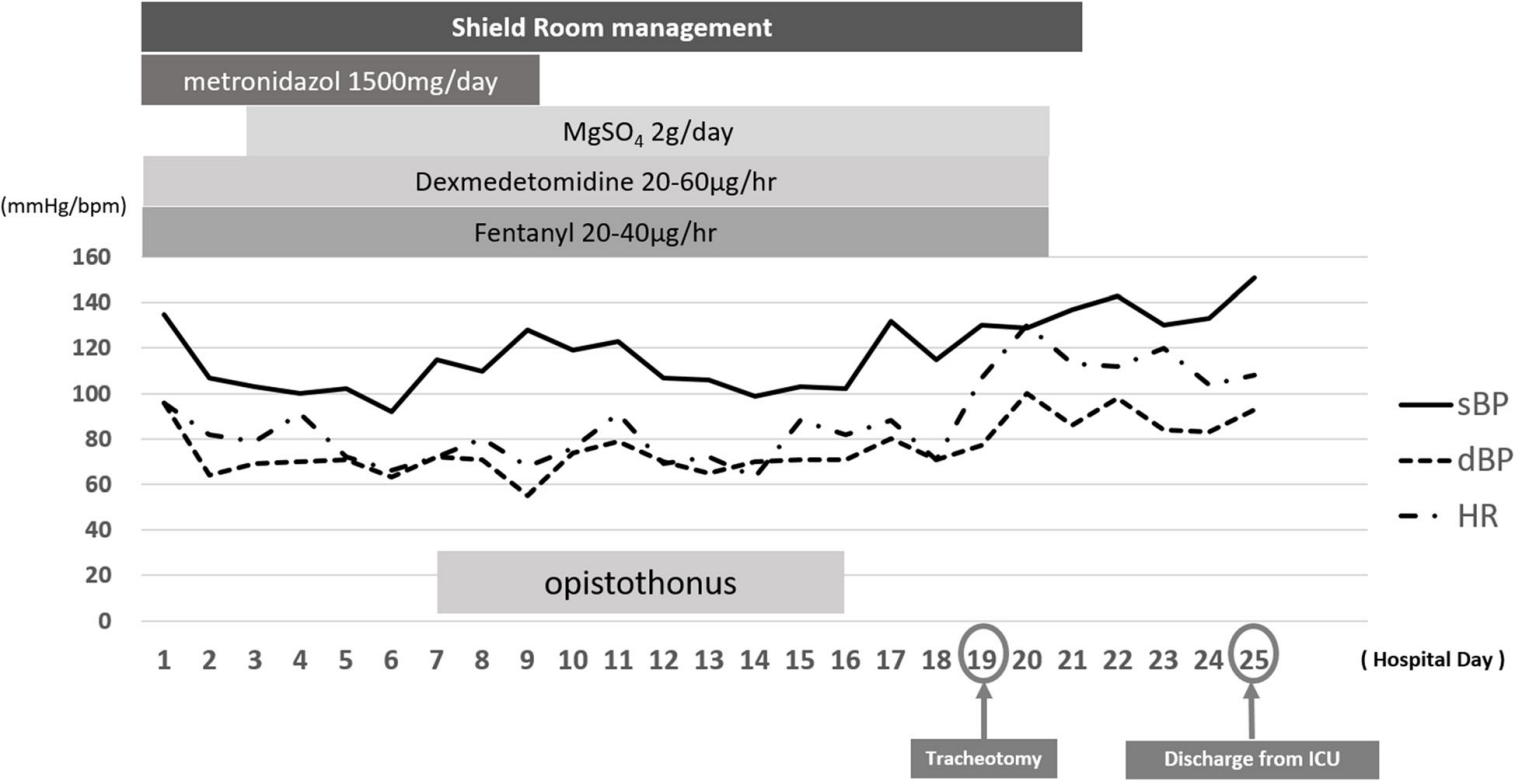
Timeline of case management from admission to discharge from the intensive care unit

An Asian woman aged over 60 years had a history of left-sided breast cancer (Fig. 1). She had received two tetanus vaccinations: first as an infant and second at around 10 years of age. She underwent cancer resection in her 40s; however, the cancer recurred and was initially treated with chemotherapy until the patient declined further treatment and stopped attending her hospital appointments. After several years, as she was no longer a patient, her medical records were discarded, leading to the loss of clinical data, including her breast cancer histopathological findings and information on disease stage and treatment. At the time of recurrence, the patient presented with an untreated breast cancer that had invaded the skin and formed a sizable tumor and ulcer in the precordial region. The patient attempted to treat her own wounds without visiting a hospital. Approximately a week before hospitalization, the patient presented with trismus. As trismus progressed, she visited a dental clinic, where the severity of her trismus precluded treatment. The patient had no disease that caused dental trismus but a cervical flexion disorder and was therefore suspected of having tetanus and subsequently referred to the Emergency Department of our hospital on the same day.

During admission, the patient was awake and alert; however, she could open her mouth only to the width of a single finger. In addition, she was unable to flex her neck due to involuntary overextension and pain in the posterior cervical muscles. Her blood pressure was 155/109 mmHg, heart rate was 120 beats/min, respiratory rate was 16 breaths/min, body temperature (axillary) was 36.4 °C, and SpO_2_ was 99% (room air). After excluding other conditions that could account for the presenting symptoms, the patient was diagnosed with tetanus.

**Fig. 2:**
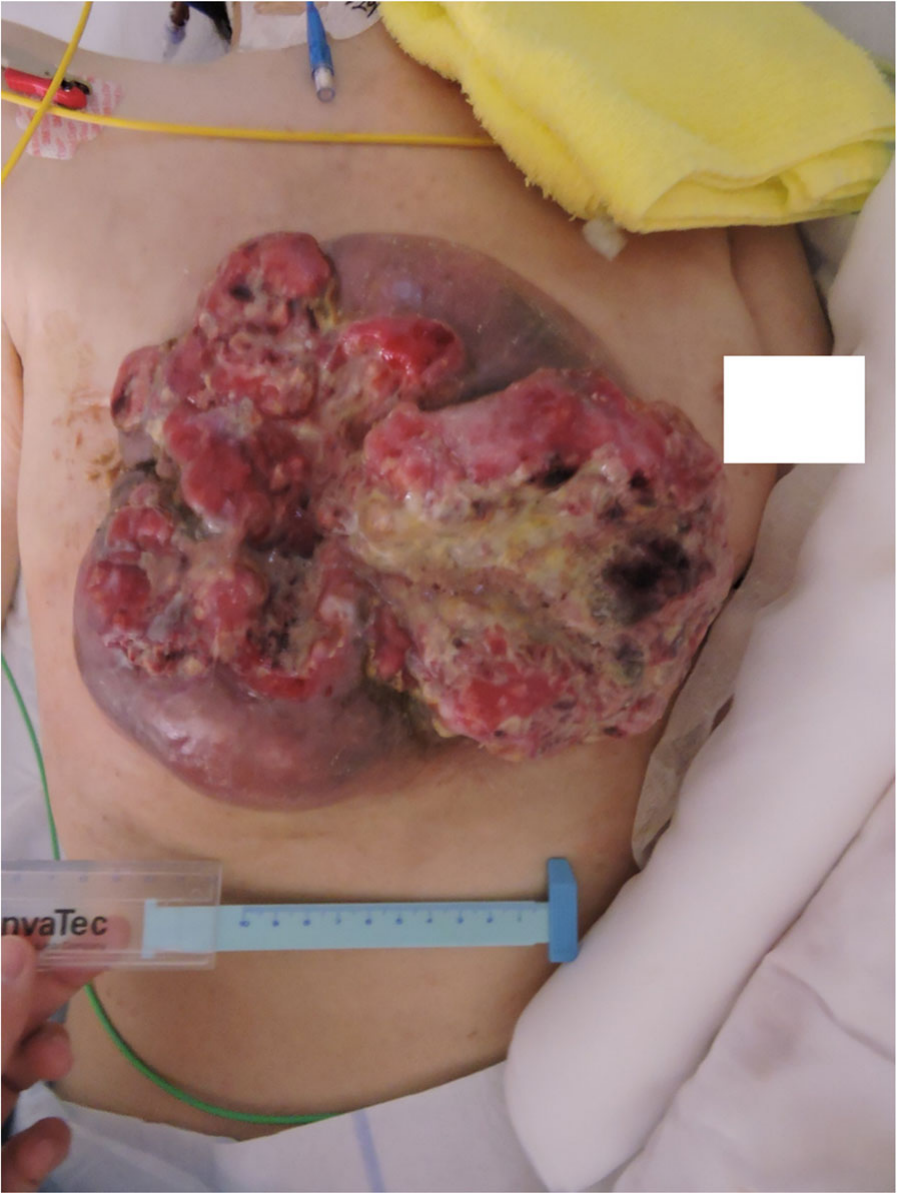
Front view of the breast cancer skin ulcer on Day 4 of hospitalization. The tumor was approximately 20 × 20 cm in size with extensive necrosis. The image is anonymized to protect the patient’s privacy

**Fig. 3:**
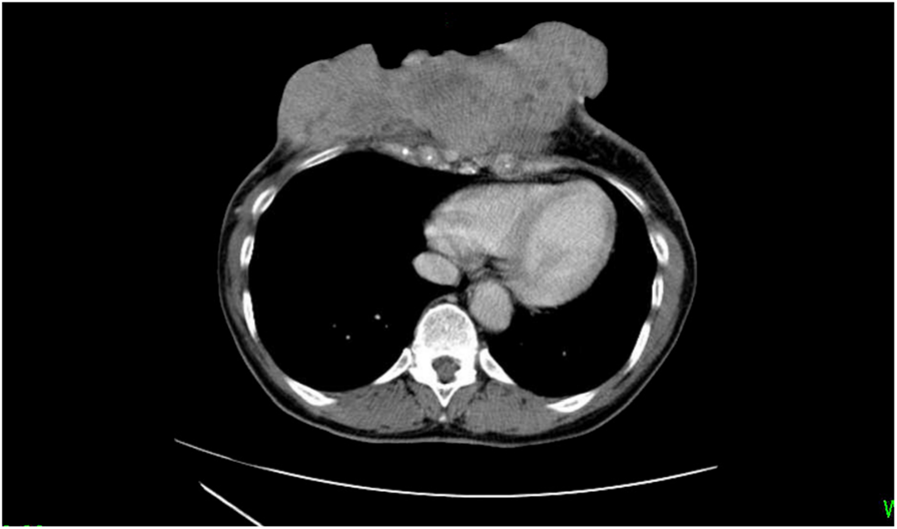
Computed tomography performed on the day of admission showing that the tumor had invaded the rib cage

Advanced breast cancer in the precordial region was suspected to account for the presence of a large tumor and skin ulcer (Fig. 2). Blood tests revealed a mild increase in the levels of inflammatory reaction parameters, likely occurring due to the skin ulcer. Computed tomography revealed no obvious cranial abnormalities; however, it showed that the precordial tumor had invaded the rib cage. Contralateral axillary lymph node metastasis was also observed; however, there was no evidence of distant organ metastases (Fig. 3). Since there was no history of minor or major trauma during 1 month prior to symptom onset, the skin ulcer was considered the most likely site of infection. As the patient reported being an avid gardener, she was at high risk of exposure to tetanus bacteria that may be present in the soil. The skin ulcer was extensive in width and depth; thus, highly invasive thoracoplasty was considered required to achieve sufficient debridement. However, progressing tetanus could cause postoperative complications such as flap necrosis. As the patient refused to consent to surgery, we did not perform surgical debridement but simply provided medical treatment.

On the first day of hospitalization, the patient received an intramuscular injection of tetanus toxoid and an intravenous injection of tetanus immunoglobulin at a dose of 4500 U. After the patient was admitted to the intensive care unit (ICU), tracheal intubation and deep sedation were performed; in addition, artificial respiration management was performed in a room that was sound- and light-shielded. Concurrently, the patient received an infusion of metronidazole at a dose of 500 mg every 8 h. Fentanyl and dexmedetomidine were administered by continuous intravenous infusion for analgesia and sedation. Magnesium sulfate was intravenously injected at 2 g/day to control muscle rigidity and convulsions.

On Day 7, the patient presented with mild opisthotonus. However, there was no significant change in the autonomic nervous system function to suggest autonomic hyperactivity.

We assessed the severity of her tetanus using the Ablett classification [22]; all symptoms were in the moderate category.

On Day 16, no sign of opisthotonus was observed, suggesting that the patient had entered a recovery period. Tracheotomy was performed on Day 19 to relieve the remaining grinding trismus. On Day 20, sound insulation and shading and sedation management were completed. Oral intake and rehabilitation commenced on Day 22. The patient was discharged from the ICU on Day 25. On Day 33, the tracheal cannula was removed, and the tracheostomy was closed.

Wound treatment consisted of daily cleaning with soap and application of the petroleum jelly Vaseline. A wound care specialist nurse instructed the patient on wound care.

On Day 45 post-tetanus onset, the patient received a second dose of tetanus toxoid. The patient was discharged on Day 54. Three years after she left the hospital, she underwent an excision of a breast cancer skin ulcer. At present, 5 years post-discharge, the patient resides at home and has shown no evidence of tetanus recurrence or long-term adverse events. At the time of writing (July 2020), the patient continues to receive chemotherapy.

## Discussion and conclusion

*C. tetani* grow and germinate in an anaerobic environment (necrotic tissue) and produce toxins. Although antibacterial agents tend to be effective in killing germinated tetanus bacteria, their effectiveness in killing these bacteria in the spore state is reduced; thus, treatment of tetanus in the necrotic tissue requires an approach that includes elements other than an antibacterial agent alone. An ulcer resulting from advanced breast cancer tends to move toward the surface of the body, exposing the chest wall in the process. This lesion is associated with anaerobic infection;

cases of tetanus infection from a breast cancer skin ulcer have been reported among other types of anaerobic infection.

The spore-forming *C. tetani* are resilient and can survive for a long time in an environment that is unsuitable for their activity. However, when the environment does become favorable, the host develops tetanus due to bacterial germination and the production of *C. tetani* toxins. Furthermore, a previous experimental study examining the impact of inoculating cancer-bearing animals showed that *C. tetani* spores that invade through a fresh wound can quickly migrate to and colonize necrotic tissues of cancer, leading to the development of tetanus [19]. In essence, a breast cancer skin ulcer functions as a reservoir of *C. tetani* toxins, even if they originally developed elsewhere.

Chronic wounds other than breast cancer skin ulcers can also be an entry point for tetanus bacteria. Barogui et al. reported a case of tetanus infection from a Bruli ulcer, which is an endemic disease in the tropics. The patient was cured of tetanus via debridement performed for the treatment of a Bruli ulcer [23]. Evidence suggests that active debridement of the infection site is important for the treatment of tetanus [1, 3, 19–21].

Recent studies have reported 15 cases of tetanus in patients with breast cancer skin ulcer (Table 1) [4–17]. Twelve of these cases (A-L) underwent surgical debridement. Two cases (C, I) died of tetanus after debridement was performed. One case (G) recovered from tetanus, but the patient died of sequela. Overall, this evidence suggests that tetanus originating from breast cancer skin ulcers is associated with a high risk of death and recurrence. As in the present case, debridement itself can be life threatening, resulting in patients refusing consent to surgery. In such cases, alternatives to debridement should be considered to help achieve good patient outcomes.

**Table 1:** Summary of previously reported cases of tetanus associated with breast cancer-related skin ulcer. No previous studies reported long-term antimicrobial therapy for the prevention of tetanus recurrence

Case	Age	Debridement	Antibiotics	Tetanus immunoglobulin	Toxoid (after tetanus infection)	Autonomic hyperactivity	Recurrence	Outcome	Reference number
**A**	62	Yes	Penicillin and metronidazole	N/A	One time	N/A	N/A	Survival	[4]
**B**	55	Yes	Yes (specifics unknown)	Yes	N/A	No	No	Survival	[5]^a^
**C**	53	Yes	Penicillin, ceftazidime, imipenem, and gentamycin	Yes	One time	Yes	No	Deceased	[6]
**D**	46	Yes	Penicillin	Yes	N/A	Yes	N/A	Survival	[7]
**E**	59	Yes	Penicillin	Yes	One time	N/A	N/A	Survival	[8]
**F**	75	Yes	Piperacillin	Yes	N/A	Yes	N/A	Survival	[9]
**G**	56	Yes	N/A	Yes	N/A	N/A	N/A	Deceased	[10]
**H**	55	Yes	Penicillin	Yes	N/A	Yes	N/A	Survival	[11]
**I**	60	Yes	N/A	N/A	N/A	Yes	No	Deceased	[12]
**J**	60	Yes	Penicillin	Yes	Two times	Yes	N/A	Survival	[13]
**K**	67	Yes	Penicillin	Yes	One time	Yes	No	Survival	[14]
**L**	65	Yes	Penicillin, metronidazole, and cefepime	Yes	One time	Yes	No	Survival	[15]
**M**	50s	No	Meropenem	Yes	No	Yes	No	Deceased	[16]^a^
**N**	40	No	Metronidazole	Yes	One time	Yes	Yes	Deceased	[17]
**O**	54	No	Metronidazole, piperacillin/tazobactam, and ceftriaxone	Yes	One time	No	Yes	Survival	[17]

^a^We contacted the authors of these papers directly and included the information they provided in this table

Three cases (M-O) of skin ulcer-related tetanus treated without debridement were previously reported. Among them, one patient (M) died as a result of tetanus, while the remaining two patients (N, O) achieved remission. However, both patients experienced recurrence: one patient (N) died of recurrent tetanus and the other patient (O) experienced tetanus remission and recurrence at least twice, but the patient’s subsequent course is unknown. Autonomic hyperactivity due to tetanus was not seen in the first remission of either case (N, O). At the time of recurrence, one of the patients (N) died due to autonomic hyperactivity, whereas the other patient (O), whose tetanus was localized, subsequently went into remission.

The severity of tetanus depends on the amount of bacterial toxin present. The most serious cases of tetanus show autonomic symptoms [24].

Severe tetanus is associated with poor prognosis compared to that associated its mild form [1, 24]. Although toxin levels may determine patient prognosis, no previous study has examined this association.

The amount of toxins present in the body is associated with the severity of tetanus and the amount of time that passes between bacterial invasion and treatment commencement. In the present case, we observed rapid remission with no recurrence. There are two reasons that may account for this finding. First, the administration of metronidazole might have killed all the tetanus bacteria before they were able to sporulate further. Concurrently, the administration of antitoxin antibodies might have neutralized the activity of the remaining toxin. However, as similar cases (M, N) have previously shown, tetanus bacillus can remain latent in the body and may cause tetanus recurrence. In animal studies in which tetanus bacteria were inoculated into cancer-bearing rats, tetanus bacteria were established in the tumor within 48 h [19]. In the present case, the patient presented more than a week after the onset of disease; the delay made it difficult to eliminate all tetanus bacteria and toxins with the use of antimicrobials and antitoxin antibodies. Second, the present patient may have acquired active immunity through two vaccinations, which may in turn have inhibited the recurrence of tetanus. The present case differs from cases N and O in that there has been no recurrence for at least 3 years after remission. Our patient had a booster shot before discharge from our hospital, while cases N and O received toxoid only once at the time of admission. Antibody titers rarely rise sufficiently with a single inoculation of toxoid. In fact, some cases of tetanus that recurred after vaccination involved antibody titers below the onset arrest concentration of 0.01 IU/ml [2, 25, 26].

Surgical resection of chronic wounds is the most reliable method of reducing the risk of tetanus recurrence. In cases where surgery cannot be performed, active immunity is required to suppress recurrence. The present patient did not experience tetanus recurrence for 3 years since the completion of treatment likely due to acquired active immunity. Tetanus can be a life-threatening disease whose occurrence can be prevented by vaccination. Cancer patients with lesions susceptible to tetanus should be actively vaccinated [3].

Nevertheless, a case of malignant tumor-associated tetanus, in which three inoculations of toxoid failed to activate immunity, likely due to the effect of cancer-induced immunodeficiency, has been reported [27]. At present, these is no clear evidence on how many toxoid inoculations should be given to cancer-bearing patients. Therefore, tetanus antibody titers should be measured in conjunction with vaccination administration to ensure that adequate antibody titers are maintained.

The tetanus antibody titer value required to prevent tetanus or maintain its remission in cancer patients remains unclear. In fact, no previous study has investigated the optimum dose, frequency, or duration of toxoid inoculation required for such patients. Further research is required to elucidate these parameters.

In summary, tetanus with chronic skin ulcer without autonomic hyperactivity can be ameliorated without debridement. However, as tetanus is prone to recurrence, the acquisition of active immunity is essential to prevent recurrence after remission. Multiple doses of vaccination may be required to ensure adequate antibody titers and immunity to tetanus are achieved in cancer patients [17, 25–27].

## Data Availability

The datasets used and/or analyzed during the current study available from the corresponding author on reasonable request.

## Abbreviation

ICU: Intensive care unit

## References

[1] Farrar JJ, Yen LM, Cook T, Fairweather N, Binh N, Parry J (2000). Tetanus. J Neurol Neurosurg Psychiatry.

[2] Birch TB, Bleck TP, Bennet JE, Dolin R, Blasser MJ (2019). 244 Tetanus (Clostridium tetani). Mandell, Douglas, and Bennett's Principles and Practice of Infectious Diseases Ninth Edition.

[3] Farnworth E, Roberts A, Rangaraj A, Minhas U, Holloway S, Harding K (2012). Tetanus in patients with chronic wounds – are we aware?. Int Wound J.

[4] Bouffet E, Pirollet B, Gaussorgues P (1986). Le néoplasme mammaire, porte d'entrée inhabituelle du tétanos. Presse Méd.

[5] Nishimura Y (1991). A case of tetanus infected by breast cancer [Translated from Japanese]. J Jpn Surg Assoc.

[6] Toh HC, Tiam AP (1997). Severe tetanus in a patient with ulcerating inflammatory breast carcinoma. A case report and review of management. Acta Oncol.

[7] Wakiyama S, Nagaie T, Maehara S, Shiroshita H, Yamashita T, Tsugawa K (1999). A case of severe tetanus caused by advanced breast cancer with infectious ulcer lesion [Translated from Japanese]. J Jpn Surg Assoc.

[8] Yip CH, Leong CM, Wahid I, Abdullah MM (2000). A rare case of breast cancer presenting as tetanus. Breast.

[9] Hamada T, Katori K, Nitahara K, Shiratake T, Kaneko T, Higa K (2001). Anesthetic management of a patient with tetanus using epidural anesthesia [Translated from Japanese]. Jpn J Anesthesiol.

[10] Fujihara T, Hase K, Yoshioka A, Kamei T, Nakamura T (2004). Anesthesia experience of breast cancer patients infected with tetanus [Translated from Japanese]. Jpn J Anesthesiol.

[11] Yamashita S, Karashima R, Tsushima A, Tawara S, Kawahara K (2007). Tetanus caused by ulcerated giant phyllodes tumor. Breast J.

[12] Wijesuriya SRE, Wijesuriya MTW, Perera MTPR, De Zylva STU, Deen KI (2007). Locally advanced breast cancer as a possible portal of entry in a patient with tetanus. Ceylon Med J.

[13] Taniyama D, Yamamoto R, Uwamino Y, Kitahara M (2013). A case of tetanus originating from ulcerated breast cancer. J J A Inf D.

[14] Liu WC, Liao GS, Yu JC, Dai MS (2015). Lethal tetanus infection following cytotoxic chemotherapy for advanced breast cancer. Eur J Oncol.

[15] Hamaya H, Kinugasa S, Okazaki T, Shinohara N, Shishido H, Takano K (2018). A case of severe tetanus with self-destructing breast cancer [Translated from Japanese]. J Jpn Soc Intensive Care Med.

[16] Harada A, Ito T, Sugano M, Hattori Y (2008). A case of tetanus infection from locally advanced breast cancer [Translated from Japanese]. J Jpn Surg Assoc.

[17] Bautista JEC, Shiong Shu LL, Pascual JLR, Pasco PMD (2015). Recurrent tetanus in diagnosed breast cancer patients of the Philippine general hospital. Acta Medica Philippina.

[18] Petrova V, Annicchiarico-Petruzzelli M, Melino G, Amelio I (2018). The hypoxic tumour microenvironment. Oncogenesis..

[19] Malmgren RA, Flanigan CC (1955). Localization of the vegetative form of clostridium tetani in mouse tumors following intravenous spore administration. Cancer Res.

[20] Prevention and management of wound infection. Department of Violence and Injury Prevention and Disability: World Health Organization; 2013. https://www.who.int Accessed 1 May 2020.

[21] Campbell JI, LTM Y, Loan HT, Diep TS, TTT N, NVM H (2009). Microbiologic characterization and antimicrobial susceptibility of clostridium tetani isolated from wounds of patients with clinically diagnosed tetanus. Am J Trop Med Hyg.

[22] JJL A (1967). Analysis and main experiences in 82 patients treated in the Leeds Tetanus unit. Symposium on Tetanus in Great Britain.

[23] Barogui YT, Simultaneously SYP-RDA (2016). Images in clinical tropical medicine. Am J Trop Med Hyg.

[24] Brook I (2007). Tetanus. Anaerobic infection.

[25] Vakil BJ, Mehta AJ, Tulpule TH (1964). Recurrent tetanus. Postgrad Med J.

[26] Bhatt AD, Dastur FD (1981). Relapsing tetanus (a case report). Postgrad Med J.

[27] Wakasaya Y, Watanabe M, Tomiyama M, Suzuki C, Jackson M, Fujimuro M (2009). An unusual case of chronic relapsing tetanus associated with mandibular osteomyelitis. Intern Med.

